# Heart Rate Turbulence: A Review

**Published:** 2003-01-01

**Authors:** Mari A Watanabe

**Affiliations:** Institute of Biomedical and Life Sciences, West Medical Building, Glasgow University, Glasgow G12 8QQ, UK and Section of Cardiology, Division of Cardiovascular and Medical Sciences, Royal Infirmary, Glasgow University, Glasgow G31 2ER, UK

Heart rate turbulence (HRT) is a recently coined phrase that describes the short term fluctuation in sinus cycle length that follows a ventricular premature complex (VPC). Its proven clinical significance lies in its ability to predict mortality and sudden cardiac death following myocardial infarction, although small studies suggest that it is also applicable to many other cardiac diseases. This review will attempt to summarize the literature to date, and to speculate on possible mechanisms. Because HRT is a new field, there are only a handful of full length papers on it. In order to present the full breadth of the research being carried out, the information provided here is based on conference abstracts as well as peer reviewed articles, and readers should keep this in mind. Most of the literature cited here, and downloadable HRT calculation programs (in C++) are available on the website  http://www.h-r-t.org

## How to compute HRT parameters

**Figure 1:**
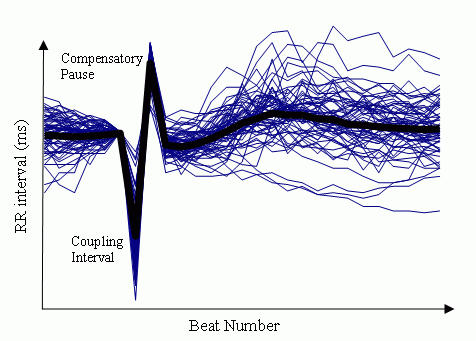
Example of RR interval sequences in one patient (thin lines), all aligned at the VPC. The thick line shows the average of the 60 sequences. Figure supplied courtesy of Dr. Georg Schmidt.

After a VPC, heart rate increases for 1 or 2 beats, then decreases. This phenomenon was discovered by Georg Schmidt's research group in Munich, when they averaged the RR interval sequences flanking VPCs (Figure 1). A rough idea of the amount of RR interval increase that can be seen is given in a study of 15 patients[1]. In that study, RR interval started to increase at beat 3 after the paced VPC, and reached a peak bradycardia of 77 ± 111 ms (m ± sd) over the baseline RR at beat 8 . HRT quantifies these heart rate changes by 2 parameters, turbulence onset (TO) and turbulence slope (TS). Roughly speaking, TO is the *amount* of sinus acceleration following a VPC, TS is the *rate* of sinus deceleration that follows the sinus acceleration. Figure 1 shows pictorially how HRT is measured. RR intervals are plotted vs beat number, with 2 beats preceding and 20 beats succeeding the VPC beat and compensatory pause. TO (%)= 100 x {(RR[1] + RR[2]) - (RR[-3] + RR[-2])} / (RR[-3] + RR[-2]), where the numbers in brackets denote beat number, with the compensatory pause being beat 0. To obtain TS (ms/beat), the slopes of RR change are calculated by fitting each 5 beat RR sequence following the compensatory pause (RR[1]~RR[5], RR[2]~RR[6], … , RR[16]~RR[20]) with a straight line. The maximum of the 16 slopes is taken to be TS. TO<0 and TS>2.5 are considered normal, TO ≥ 0 and TS ≤  2.5 are considered abnormal. In other words, strong sinus acceleration followed by rapid deceleration marks a healthy response. In the seminal HRT publication by Schmidt et al[2], the 24 RR intervals encompassing each VPC (2 before the VPC, 20 after the compensatory pause) were averaged over a 24 hour Holter record. HRT was calculated for this averaged response, such that each patient was represented by one value each for TS and TO.

Measurement of HRT has not been standardized yet. Some of the recent studies from the Schmidt laboratory exclude RR sequences that don't have at least 3 or 5 sinus intervals before the VPC, but have relaxed the criteria for the minimum number of post-compensatory pause sinus RR from 20 to 15 [3,4]. With regard to averaging of RR, HRT can also be calculated for each VPC, then averaged, to give a mean and standard deviation of HRT parameters for a given individual [5]. For reasons not yet clear, perhaps due to improvement in signal/noise ratio by the process of averaging as suggested by Figure 1, the original method of averaging the RR values before HRT calculation provides better prediction of mortality, and is to be recommended whenever circumstances permit. HRT can also be averaged for groups of VPCs satisfying selected criteria, such as normalized coupling interval [3].

**Figure 3:**
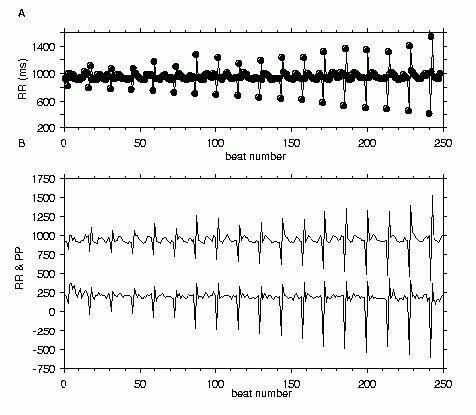
Induced HRT. ***Panel A***. RR intervals from a patient undergoing clinical electrophysiology study for supraventricular tachycardia. A stimulus was delivered at the RV apex after 20 sinus beats at various coupling intervals shorter than the baseline RR interval. Only the RR for the 2 beats before and 10 beats after the extrastimulus and compensatory pause are shown for each extrastimulus. ***Panel B***. The RR data from panel A and pulse pressure (systolic - diastolic blood pressure) for the same beats are shown together. The pulse pressure was multiplied by 10 and shifted down by 800 mmHg so as not to overlap with the RR data.

Analysis of HRT is not limited to Holter records. HRT analysis has been conducted on event records from implanted cardiac defibrillators [5]. HRT can also be induced by intracardiac pacing in the electrophysiology lab [1,6-8] or in patients with implanted cardiac defibrillators [9]. Such HRT has been called *induced HRT* [6] to distinguish it from that measured from Holter recordings. Figure 3A shows an example of normal induced HRT. The stimulation protocol suggested for measuring induced HRT is to compute TS and TO from the average of RR sequences following 10 extrastimuli given with a spacing minimum of 20 sinus beats, and a coupling interval that is 60 ~ 70% of sinus cycle length [6].

## HRT as a predictor of mortality

The first full length publication introducing HRT and the 2 parameters quantifying it was Schmidt et al's 1999 Lancet article (hereafter referred to as the Lancet study) [2]. It was preceded by an abstract, in which HRT was called by its original name of heart rate chronotropy [10]. In the Lancet study, Holter recordings from one hundred survivors of acute myocardial infarction with frequent VPCs were used to determine the discriminating threshold between normal and abnormal HRT values (TS=2.5, TO=0%). The recordings were made at least 3 months after the acute myocardial infarction. These thresholds were blindy (and therefore prospectively in that sense) applied to Holter records from a total of 1191 patients from 2 large post-myocardial infarction clinical trial groups, the placebo arm of the European Myocardial Infarction Amiodarone Trial (EMIAT n=614), and the Multicentre Post Infarction Program (MPIP n=577). The Holter recordings were made in the 2nd or 3rd week after the index infarction. There were 162 deaths (13.6%) during the median follow-up period of 21 (EMIAT) and 22 (MPIP) months. With total mortality as the endpoint, *univariate* analysis showed that TS was the strongest risk stratifier in EMIAT (relative risk 2.7), while TS was the second strongest risk stratifier in MPIP (relative risk 3.5) after poor EF (EF<30%). A relative risk of 3 means that patients with abnormal TS are 3 times more likely to die than those with normal TS. *Multivariate* analysis showed that in EMIAT, each of TO, TS, previous infarction history, EF, and mean HR (>75) were independent predictors, and in MPIP, EF and TS were independent predictors. If one predictor is independent of another, it means that the criteria identifies a different group of patients, and that for example, the patients with high HR do not necessarily coincide with the patients with low EF. Multivariate analysis also showed that combined TS and TO was the most powerful risk stratifier in both the EMIAT (relative risk 3.2, 95% confidence interval 1.8-5.6) and MPIP (relative risk 3.2, 95% confidence interval 1.7-6.0) populations. (The *combined* parameter is considered abnormal if both TS and TO are abnormal.) Indeed, in contrast to TS and EF, none of advanced age (>65 years), previous myocardial infarction, high mean HR, heart rate variability triangular index ≤ 20, and presence of arrhythmia on Holter (>9 VPC/hour or non-sustained ventricular tachycardia of >2 beats), were independent mortality predictors in the multivariate analysis in the MPIP population.

## HRT as a predictor of cardiac arrest

Data from the Autonomic Tone and Reflexes after Myocardial Infarction (ATRAMI) has been used to study HRT as a predictor of cardiac arrest [11]. The study involved 1212 survivors of acute myocardial infarction, with a mean follow-up period of 20.3 months. Patients in this study were at lower risk compared to EMIAT in which only patients with EF<40% were enrolled, and compared to MPIP in which no patients had had thrombolytic treatment. The endpoint was cardiac arrest, both fatal and non-fatal, which was reached in 49 patients (4.0%). Univariate analysis showed that TS and combined TS and TO both produced moderately high relative risk values (4.1 and 6.9 respectively, p<0.0001 for both). A composite index of cardiac autonomic function was made from TO, TS, the SDNN parameter of heart rate variability, and baroreflex sensitivity. Patients in whom all of the factors making up the composite index were abnormal were found to be 16.8 times more likely to have a cardiac arrest than those in whom all 4 factors were normal. Unfortunately, of the 49 patients who had eventual cardiac arrest, only 5 satisfied this criterion. At the same time, there were 10 other patients in whom all 4 factors were abnormal and who did not have cardiac arrest. This brings us to the next topic.

## The issue of sensitivity

The sensitivity and positive predictive accuracy of HRT is better than other available non-invasive tests, but not much better. When TS and TO were combined, the Lancet study [2] reported a sensitivity, specificity, and positive predictive accuracy of approximately 30, 90, and 32% respectively, for both the EMIAT and MPIP data. In simpler terms, by setting TS and TO thresholds at 2.5 and 0, we catch 30% of the patients who will have events, but we also catch so many false positives that if there are 100 patients with abnormal HRT, only a third of them will be true positives. The Lancet study reported a positive predictive accuracy of 18 ~ 24% (EMIAT), and 16~30% (MPIP) for the other 6 variables of advanced age, previous myocardial infarction, high HR, low heart rate variability triangular index, presence of arrhythmia on Holter, and low EF. The positive predictive accuracy which came closest to matching combined TS and TO as a parameter was EF in the MPIP data at 30%. However, EF had better sensitivity (43%) and specificity (85%) than the combined HRT parameter.

The excellent commentary by Macfarlane accompanying the Lancet article specifically compares HRT to commonly used electrocardiographic predictors, namely, heart rate variability, late potentials, ST-T changes, QT dispersion, and prolonged QT [12]. In the commentary, sensitivity, specificity and positive predictive accuracy of low heart rate variability (SDNN< 50 ms) are given as 34, 89 and 34%, neatly matching values for combined TS and TO. The positive predictive accuracy of HRT and heart rate variability is at least 2 times greater than that of the other predictors, but the sensitivity of late potentials, for example, is much higher at 93%. How does HRT compare to baroreflex sensitivity? According to a study based on the ATRAMI data, TS had a positive predictive accuracy of 12.5% at 40% sensitivity, and thus came out ahead of low baroreflex sensitivity with its positive predictive accuracy of 7.8% [13]. Certainly, predictive accuracy can be improved by combination with other risk predictors. At 40% sensitivity, abnormal TS combined with poor heart rate variability gave a positive predictive accuracy of 20.3%, and TS combined with low EF, a positive predictive accuracy of 17.3% [13], although as mentioned 2 paragraphs before, combining tests also results in fewer patients who satisfy the criteria.

In summary, the key number to remember out of the forest of statistics concerning HRT is the number 30. TS and TO combined (when both are abnormal) give a sensitivity of 30%, and a positive predictive accuracy of approximately 30%. This is consistent with another"30" from the Lancet study [2]. When comparing patients who had 0, 1 or 2 abnormal HRT measures, the 2 year mortality rate was 9, 15, and 32% in the MPIP population, and 9, 18 and 34% in the EMIAT populations. In other words, 1 in 3 post-myocardial infarction patients whose TS and TO are both abnormal 2 weeks after their infarction, are likely to be dead in 2 years.

## HRT is not always measurable

HRT has the opposite problem from heart rate variability. Ectopic beats are always excluded from analyses of heart rate variability, and criteria exist for how much of a Holter record can be deleted before portions of it are considered invalid. In contrast, HRT obviously cannot be measured in subjects who do not have VPCs. This makes the study of HRT in healthy subjects difficult, and researchers have to resort to studying induced HRT. In the Lancet study, 138 of 715 patients (19%) in the MPIP population, and 129 of 743 (17%) in the EMIAT population had to be excluded because of absence of VPCs, atrial fibrillation, insufficient or missing Holter recordings, and missing EF data (MPIP) [2]. According to Raphael Schneider (personal communication), if one excludes patients with atrial fibrillation, approximately 10% of patients with Holter records cannot have HRT measured because of absence of VPCs. In a study in which our research group looked at 1000 beat RR data from patients with implanted defibrillators, we found that too many VPCs also resulted in exclusion of some records from study, the reason being that the 20 beat sinus RR following the compensatory pause were interrupted by VPCs, precluding measurement of TS [5]. In this high risk group, 14 of 84 (17%) control records and 9 of 84 (11%) arrhythmia records had no VPCs. A further 21 (25%) control and 19 (23%) arrhythmia records had no stretches of > 10 sinus RR following the compensatory pause. However, one should note that these are results for 1000 beat records, i.e., short 10~15 minute records, and that furthermore, a full 10 beats is frequently unnecessary to determine whether TS is normal or not, because the average record has an RR change slope that reaches a maximum for the 5 beat RR sequence RR[3]~RR[7] or RR[4]~RR[8] [5].

## Applicability of HRT to patients on beta-blockers

Most predictors for post-acute myocardial infarction mortality perform poorly in patients on beta-blockers. In a study of 591 patients in EMIAT's placebo arm, 271 patients were on beta-blockers, 320 were not [14]. In the patients on beta-blockers, combined TO and TS was found to be the only independent predictor of mortality with a relative risk of 3.8 (p=0.004). Mean HR, previous myocardial infarction and low EF which were independent predictors for patients not taking beta-blockers, all failed when applied to the patients on beta-blockers. In this regard, HRT is seen to have a distinct advantage over some of the other risk predictors.

## HRT as a risk predictor in other diseases affecting the heart

HRT has been assessed in patients with diabetes mellitus [15,16], congestive heart failure [17,18], idiopathic dilated cardiomyopathy [19-21], and Chagas disease [22]. In the earlier study of diabetes, one hundred patients with coronary artery disease were divided into 2 groups, those with and without diabetes [15]. TO was found to be a significant predictor of cardiac mortality (p<0.05), although TS was not. In a second study of diabetes using 586 patients from the placebo arm of the EMIAT, HRT parameters failed as independent predictors of mortality in the 95 patients with diabetes [16]. In a study of 199 patients with congestive heart failure (mean duration of follow-up 971 ± 378 days, median EF of 22% and 47 cardiac deaths), abnormality of both TO and TS produced the highest risk ratio of 4.1 for cardiac deaths, compared to 3.3 for presence of non-sustained ventricular tachycardia, 2.6 for abnormal baroreflex sensitivity, and 2.0 for the triangular index of heart rate variability [17]. With regard to idiopathic dilated cardiomyopathy, a small study of 12 patients showed that TS and TO were significantly worse (both p<0.01) compared to 13 healthy subjects [20]. A larger study was reported recently, involving 178 patients also with idiopathic dilated cardiomyopathy [21]. The endpoint was heart transplantation; follow-up duration was 28 ± 17 months. 21 patients required a heart transplant. Statistical analysis showed that TS, TO, and combined TS and TO were significantly worse in patients requiring transplant (p<0.05). The relative risk, however, was found to be very low, and the setting of a different threshold of HRT parameters for patients with dilated cardiomyopathy was suggested. In a comparison of 11 healthy subjects and 126 patients with Chagas disease, TS and TO values were found to be significantly different (p<0.001) [22]. These preliminary studies show that HRT is diminished not only in post-myocardial infarction patients, but those with a variety of diseases affecting the heart, and in the case of congestive heart failure and possibly diabetes, may provide clinically useful risk stratification information

## Mechanism of HRT

The gross mechanism of HRT is easy to understand. BP of a VPC beat is normally small because of several factors. Diastolic filling is incomplete leading to low ejection volume. Various membrane ion channels have not recovered fully, leading to a short action potential (electrical restitution). The low volume/stretch leads to reduced contractility (Frank Starling mechanism). Finally, the abnormal sequence of activation of the ventricles leads to a less synchronized contraction. The sudden drop in blood pressure due to all of these factors activates the aortic and carotid baroreceptors, which increase HR through the baroreflex arc. A VPC is normally followed by a compensatory pause. The BP of the compensatory pause beat is higher than BP of a sinus beat, a phenomenon called post-extrasystolic potentiation. Some of the long time constant ion channels have recovered more fully leading to a longer action potential, and intracellular calcium is greater due to mechanisms still debated. The sudden increase of BP reduces HR, again via the baroreflex arc. If HR simply tracks BP changes, the sequence of events just described explains tidily, the sinus acceleration and deceleration that is HRT. In summary, VPC → low BP → HR increase → compensatory pause → high BP → HR decrease.

A figure in a recent publication illustrates this beautifully. Voss et al show a simultaneous ECG and BP trace from a healthy subject [20]. In the ECG trace, use of a caliper shows that RR interval shortens following the VPC, then lengthens. In contrast, changes in the BP trace caused by the VPC demonstrate a striking resemblance to the HRT pattern of RR intervals (as shown in figure 1) After the compensatory pause, both systolic and diastolic BP are low for 2 beats, then gradually increase, peaking at about the 7th beat before gradually returning to normal. If this BP trace is typical of a normal response, HRT mirrors *BP turbulence* closely. Figure 3B shows the similarity of pulse pressure values to RR values during induced HRT.

In contrast to this gross mechanistic picture, the fine details of HRT's mechanism have not been established. The main unanswered question concerns sympathovagal balance. During the sinus acceleration quantified by TO, does vagus withdrawal predominate or sympathetic recruitment? What about during the sinus deceleration quantified by TS? It is important to ask this question, because answering it will give us clues as to why HRT is a cardiac arrest predictor, and how cardiac arrest might be prevented. Several lines of evidence point to a predominant vagal mechanism. The strongest of these is the fact that atropine abolishes HRT completely [7,23]. Based on an induced HRT study of 16 patients without structural heart disease, it has also been shown that intravenous esmolol doesn't alter TS or TO [24]. Thirdly, as mentioned earlier, HRT is effective as a risk predictor even in patients on beta blockers [14]. The case for a predominant sympathetic role is weaker. A mathematical model has shown beta-blockade to reduce TS, although not TO [25]. However, before drawing any conclusions, the phenomenon of accentuated antagonism must be taken into account. Sympathetic and para-sympathetic effects are not additive; taking away one limb does not leave one with the other limb, it reduces the other limb as well. For example, vagal effects are stronger in the presence than in the absence of sympathetic tone. Therefore, the importance of the sympathetic nervous system in producing a healthy HRT pattern cannot be ruled out solely on the basis of studies using atropine and beta blockers. The fact that TS and TO are independent risk predictors also suggests that HRT is not a purely vagal phenomenon. If that were the case, patients with abnormal TS and TO should overlap significantly.

Separating the sympathovagal influences on HRT is complicated by the bidirectional change in BP seen with the VPC and subsequent compensatory pause. What if no compensatory pause were present? What happens to HRT? One might expect atrial premature contractions and interpolated VPCs to form a good testing ground, because they lack a compensatory pause. The HRT characteristics of the latter have not been studied yet, but atrial premature contractions have been studied, and it appears that they evoke HRT [26,27]. A study of 227 atrial premature contractions from 10 normal subject Holter records found TS of 17 ± 1 (mean ± sem), which was significantly smaller (p<0.001) than the TS for VPCs found in the same group of subjects (26 ± 2) [27]. This suggests that the compensatory pause is irrelevant for producing HRT, with the smaller TS in the case of atrial premature contractions being due to the less dramatic fall in BP, because the sequence of ventricular contraction is preserved in atrial premature contractions. In a study that could be interpreted as leading to the opposite conclusion, Voss et al showed that HRT was much reduced and the BP of the compensatory pause beat much higher in patients with idiopathic cardiomyopathy than in healthy subjects [20]. Based on this observation, they reasoned that the high BP of the compensatory pause in the myopathy patients swamped any effects of the low BP of the VPC and therefore reduced HRT. In other words, the compensatory pause affected (reduced) HRT greatly. A possible flaw of this hypothesis is that RR variability is already low in DCM [28]. To attribute reduced HRT to high BP from the compensatory pause raises the question, what causes the reduced RR variability in the stretches of record that lack VPCs? However, the truth is, post-extrasystolic potentiation occurs even in the absence of a fully compensatory pause, and neither interpolated VPCs nor atrial premature contractions prevent the post-extrasystolic pressure increase that is probably responsible for the sinus deceleration phase of HRT.

## Effect of HR on HRT

 Practically all of the studies that have looked at the relationship between HRT and HR agree that HRT is reduced by high HR [6,29,30]. One of the larger studies involved 591 patients with suitable Holter records from the placebo arm of the EMIAT population [29]. During follow-up, 82 patients died. The 38,374 and 40,409 VPCs, respectively from the survivors and non-survivors were pooled and sorted according to the HR (calculated from the pre-VPC RR) before calculation of TS and TO. Linear regression showed correlations between HRT parameters and HR (all p<0.0001, r value range 0.65~0.93), both for survivors and non-survivors. In survivors, regression of TO on HR gave a slope of 0.044, regression of TS on HR gave a slope of -0.061. The linear regression of TO on HR in non-survivors was poor (r=0.65), because at HR above 80, TO became approximately 0 (was abolished).

A small study of induced HRT in a mix of 28 patients with supraventricular and ventricular tachycardia, similarly found a significant correlation between TS and HR when each patient was represented by one mean TS value and HR preceding the VPC (p<0.02 ), but not for TO [6]. Another study found TO to be diminished (close to 0) at high HR, leading to warnings about false positive TO at high HR [30]. Such an influence of HR on HRT raises the question of whether threshold values for normal and abnormal HRT values have to be tied to HR, similar to Bazett's formula for QTc. Schmidt's group have already reported that a parameter which they call turbulence dynamics, defined as steepness of the regression line of TS on HR, has strong predictive value for mortality [31].

## Effect of VPC coupling interval on HRT

Several studies have looked into the relationship between HRT and VPC coupling interval [3,6,8,27]. Three of these studies, (two based on Holter recordings, one based on induced HRT), found correlations of varying degrees between VPC coupling interval and HRT parameters, with shorter coupling intervals generally producing greater absolute values of TS and TO [3,8,27]. The largest of these studies involved the same 591 patients from the placebo arm of the EMIAT population as in the HR/HRT discussion above [3]. The VPCs from the survivors and non-survivors were pooled and sorted according to normalized VPC coupling interval before calculation of TS and TO. Linear regression showed correlations between HRT parameters and VPC coupling interval (all p<0.0001, r value range 0.67 ~ 0.89), both for survivors and non-survivors. In survivors, regression of TO on coupling interval gave a slope of 0.028, regression of TS on coupling interval gave a slope of -0.066, whereas the absolute value of these slopes were smaller for the non-survivors at 0.017 and -0.049 respectively. A second study focused on 171 VPCs from 10 normal subjects and found a significant correlation between TS and normalized VPC coupling interval (r=-0.4, p<0.001) [27]. In it, TS was also found to correlate significantly with atrial premature beat coupling interval. A third study measured induced HRT in 37 patients undergoing evaluation for ventricular tachycardia [8]. This study only found correlations between TS and TO and normalized coupling interval in patients with EF>40% (r=-0.61, 0.68 respectively, p<0.0001), and found no correlations between atrial premature beat coupling interval and HRT parameters.

A fourth study, also measuring induced HRT in 28 patients undergoing evaluation for supraventricular or ventricular tachycardia, found no correlations at all between VPC coupling interval and HRT parameters, regardless of whether patient data were pooled as in the 3 studies that did find correlations, or whether correlations were sought in individual patients [6]. How is it possible for one study to find no correlation at all, when 2 studies found correlations with p<0.0001? The most probable mechanistic reason is the effect of baseline heart rate. As discussed in the previous paragraph, HRT is blunted at high HR. If HR is high, then regardless of normalized coupling interval, HRT will be low and unlikely to be correlated with coupling interval. This is especially true of TO, because at HR above 80~90, TO is nearly 0, and may be the reason it is easier to show a coupling interval/TS correlation than a coupling interval/TO correlation. In the study that found no correlation, 12 of the 28 patients in the 4th study had mean HR>75 bpm. It must also be noted that in one study reporting a high correlation, VPCs with normalized coupling intervals of 100% and greater were included [8]. Because a coupling interval of 100% should diminish blood pressure very little, and therefore produce no HRT, inclusion of this *obligate* zero skews the results towards better statistical significance, because TS is generally positive and TO is generally negative.

In summary, HRT as a mortality predictor should probably be assessed at HR <80 bpm whenever possible. The effect of coupling interval as separate from HR seems debatable, and awaits further study. Plotting HRT vs both HR and non-normalized coupling interval as shown in an early abstract, may be one effective approach to answering this question [32].

## Correlation of HRT to other autonomic indicators

As might be expected, there is a strong correlation between HRT parameters and other autonomic indicators. In one study, TS and TO were found to correlate significantly with almost all heart rate variability time domain parameters, including SDNN, heart rate variability index, TINN, and RMSSD (p<0.0001 for most)  [33]. TO (but not TS) also correlated with pNN50. In another study looking at HRT parameters and baroreflex sensitivity, both TS and TO correlated moderately (r=0.44, r=-0.34 respectively) but significantly (p<0.001) with baroreflex sensitivity measured invasively (iv phenylephrine) [34]. TS has also been reported as correlating strongly (r=0.70, p<0.0001) with baroreflex sensitivity alpha-index, a non-invasive baroreflex sensitivity measure [34]. Finally, in line with what is known about the effects of age and gender on other autonomic indicators [35], HRT diminishes with age [36], and is greater in women [36].

## AV nodal turbulence

If a VPC affects sinus rate through baroreflexes, it is probable that AV conduction is affected as well. Two studies have looked at this. The first measured the 20 AA and VV intervals following right ventricular extrastimuli and plotted one against the other. All points lay cleanly on a line of slope 1 (r^2^= 0.999), indicating no effect of VPCs on AV nodal conduction time [23]. A very recent study did find an effect [37]. In that study, patients with normal induced HRT patterns (14/26) in their RR intervals also exhibited AV conduction delay turbulence. Compared to RR interval turbulence, AV delay turbulence onset was barely existent (-0.6 ± 3.4%), and AV delay turbulence slope was 0.9 ± 1.3 ms/beat, clearly smaller than TS of RR interval (14.7 ± 16.5). The pooled RR and AV delay vs beat number profiles showed a 6% difference between maximum and minimum RR, and a difference < 2% for AV delay. It is probable that just as ventriculophasic modulation of AV nodal conduction is masked by opposing effects of ventriculophasic modulation of the sinus rate [38], increases in PP interval prevent AV nodal conduction delay from becoming manifest in the case of HRT.

## Novel HRT measures

In addition to TS and TO, there are various HRT parameters that have been proposed and studied. *Turbulence dynamicity* was already defined in the HRT/HR section [31]. *Turbulence frequency decrease* is a parameter that measures HRT in the frequency domain [39]. This parameter is obtained by fitting a sine wave equation to the post-compensatory pause RR values with a frequency term that decreases over time. It appears to contain mortality information independent of the time domain parameters TS and TO [39]. *Turbulence timing* is a parameter that notes the first beat number of the 5 RR interval sequence where TS is found (where the slope of RR change is maximum) [5,6]. Turbulence timing is dependent on HR, and the relationship indicates that the sinus deceleration phase of HRT is earlier at slow heart rates [6]. *Turbulence jump* is a parameter which quantifies the maximum difference between adjacent RR intervals [9]. It was found to predict recurrence of ventricular tachycardia and fibrillation in patients with dilated cardiomyopathy. *Correlation coefficient of TS* is the correlation coefficient of the regression line fitted to the 5 RR intervals giving the maximum slope (i.e., where TS is defined) [40]. It is an independent predictor of mortality in post-infarction patients, but its risk ratio is lower than that for TS or TO. Further studies are needed to see if any of these parameters provide significantly superior risk stratification to TS and TO, given that TS and TO are simpler to measure than some of these parameters, and can be measured in short records.

## Summary

Despite its limitation, namely, the fact that one cannot measure HRT in patients with atrial fibrillation and patients lacking VPCs, HRT is a simple and elegant way of measuring cardiac autonomic function in patients at risk. It is nature's own autonomic perturbation experiment, and is non-invasive. Compared to other non-invasive risk predictors, the relative risk and positive predictive accuracy of HRT are only modestly better, but the ease with which it can be measured, and its applicability as a predictor in patients on beta-blockers make HRT significantly more attractive. One might even instruct general practitioners to see if RR interval after a random VPC on an electrocardiogram increases by at least 12 ms from the shortest to longest RR within 10 beats, as a lazy man's HRT. Such conjecturing aside, if ways can be found of increasing HRT's positive predictive accuracy, we may be one step closer to deciding who needs to get an automatic implantable defibrillator in a cost effective way. With such obvious promises in store, HRT is being considered for inclusion in a large scale ICD implantation trial. This author, for one, has high expectations for HRT's future.

## Figures and Tables

**Figure 2:**
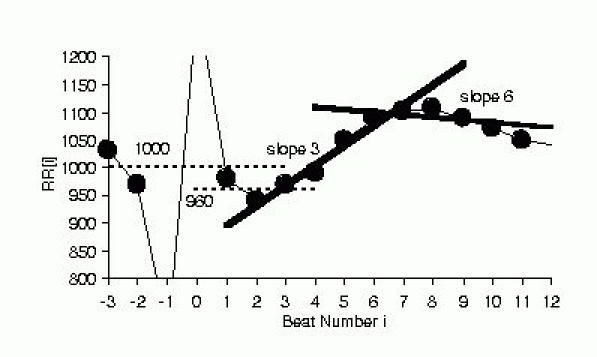
Schematic figure showing measurement of TO and TS. TO is the difference in the average of the 2 RR intervals preceding and succeeding the VPC/compensatory pause sequence expressed as a percentage. In the example shown, TO= - 4% from 100 x (960-1000)/1000. TS is the maximum of regression slopes computed for 5 consecutive RR sequences. In the example, the regression lines for beats 3~7 (slope 3) and beats 6~10 (slope 6) are shown, and TS was 36.4 because slope 3 had the largest slope of 36.4 ms/beat number among slopes 1 through 16.

## Glossary of abbreviations

BP: blood pressure
EF: left ventricular ejection fraction
HR: heart rate
HRT: heart rate turbulence
TO: turbulence onset
TS: turbulence slope
VPC: ventricular premature contraction
ATRAMI: autonomic tone and reflexes after myocardial infarction
EMIAT: european myocardial infarction amiodarone trial
MPIP: multicentre post-infarction program
NN: normal QRS to normal QRS interval
pNN50: proportion, NN50/NNtotal
RMSSD: root of mean squared differences of NN
SDNN: standard deviation of NN
TINN: triangular interpolation of NN histogram
